# Chronic Rhinosinusitis With Nasal Polyps Successfully Treated With Tezepelumab After an Inadequate Response to Dupilumab: A Case Report

**DOI:** 10.7759/cureus.96961

**Published:** 2025-11-16

**Authors:** Ryo Wakasugi, Takeshi Takahashi, Takanobu Sasaki, Toshiyuki Koya, Arata Horii

**Affiliations:** 1 Otolaryngology - Head and Neck Surgery, Niigata University Hospital, Niigata, JPN; 2 Respiratory Medicine and Infectious Diseases, Niigata University Hospital, Niigata, JPN

**Keywords:** chronic rhinosinusitis with nasal polyposis, dupilumab, innate lymphoid cell group 2, tezepelumab, thymic stromal lymphopoietin

## Abstract

Chronic rhinosinusitis with nasal polyps (CRSwNP) is a type 2 inflammatory disease that is often refractory to surgery and requires treatment with biologics, including dupilumab (DUP), a monoclonal antibody targeting IL-4/13. We report a case of severe CRSwNP with comorbid severe asthma that showed unfavorable outcomes with DUP treatment but markedly responded to tezepelumab (TEZ), a monoclonal antibody targeting thymic stromal lymphopoietin (TSLP).

A 41-year-old man with steroid-dependent intractable asthma and CRSwNP presented with anosmia and nasal obstruction. Although the patient had undergone functional endoscopic sinus surgery (FESS) and subsequently received systemic corticosteroids (SCSs) and DUP therapy (prescribed by respiratory medicine clinicians), the nasal obstruction and anosmia persisted, and nasal polyps continued to enlarge. Owing to poor control of asthma, the Department of Respiratory Medicine switched the systemic therapy to TEZ. Four months after TEZ initiation, asthma control improved along with reductions in the nasal polyp score (NPS) and computed tomography scores. Notably, olfaction improved from anosmia to mild hyposmia within one year. SCSs were successfully discontinued without asthma exacerbation.

TSLP, which acts upstream of IL-4/13 in the type 2 inflammation cascade, may be a promising therapeutic option for patients with CRSwNP and comorbid asthma refractory to DUP. Further studies are warranted to validate the efficacy of TEZ in the management of CRSwNP.

## Introduction

Chronic rhinosinusitis with nasal polyps (CRSwNP) is characterized by a diffuse bilateral anatomic distribution dominated by type 2 inflammation. Functional endoscopic sinus surgery (FESS) is the standard of care for CRSwNP. However, the underlying refractory nature of the disease necessitates postoperative adjunctive medical treatments in most patients, including systemic corticosteroids (SCSs). Long-term or repeated SCS use is associated with an increased risk of acute and chronic adverse effects [1].

Biologic therapies targeting type 2 inflammation have emerged as promising treatment options for CRSwNP. Dupilumab (DUP), an anti-IL-4 receptor alpha monoclonal antibody, is the most effective and widely used biologic in Japan. Its application is also supported by the European Position Paper on Rhinosinusitis and Nasal Polyps 2020 [1]. Despite its efficacy, some patients remain unresponsive to or are ineligible for DUP treatment owing to eosinophilia, thereby necessitating the development of potential alternatives. In a previous cohort study of 80 patients, 72.5% were responders and 27.5% were nonresponders to DUP [2].

Tezepelumab (TEZ), an anti-thymic stromal lymphopoietin (TSLP) monoclonal antibody, has demonstrated broad anti-inflammatory effects in severe asthma, including reduced exacerbations and type 2 biomarkers, regardless of the baseline eosinophil level [2]. A recent phase 3 trial suggests its potential utility in CRSwNP [3]. In this article, we report a case of CRSwNP with comorbid severe asthma that responded inadequately to FESS, SCSs, and DUP but showed significant improvement after initiating TEZ, with olfaction improving from anosmia to mild hyposmia at one year.

The objective of this study was to report clinical, endoscopic, radiologic, and olfactory outcomes in a patient with CRSwNP and comorbid severe asthma who exhibited an inadequate response to FESS, SCSs, and DUP but improved after switching to TEZ.

## Case presentation

A 41-year-old man with a long history of asthma was being treated with combination inhaled therapy (long-acting β₂-agonist/long-acting muscarinic antagonist/inhaled corticosteroid), theophylline, montelukast, and fexofenadine; however, his asthma remained poorly controlled, necessitating repeated hospital admissions for intravenous steroid infusions and SCS bursts, fulfilling the Global Initiative for Asthma criteria for severe asthma [4]. For comorbid CRSwNP, he had been using an intranasal corticosteroid spray (fluticasone furoate).

**Figure 1:**
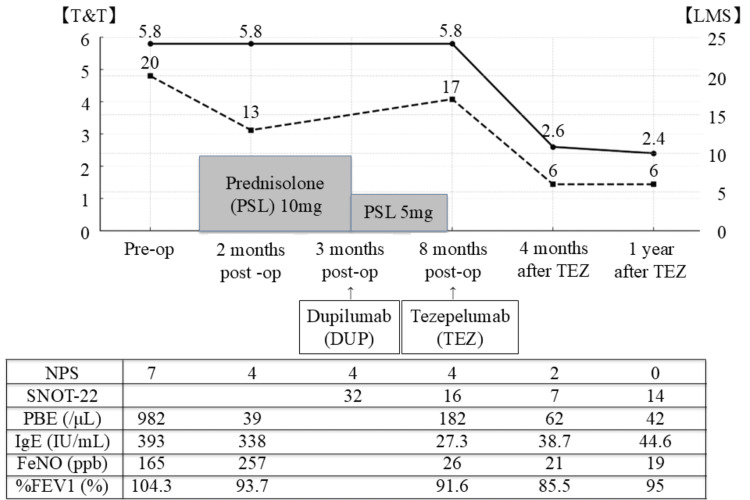
Overview of chronological changes in treatments, as well as upper and lower airway biomarkers To treat severe asthma, systemic therapies, SCS, DUP, and TEZ were selected and managed by the Department of Respiratory Medicine clinicians; the otolaryngology team performed FESS and provided sinonasal follow-up. Despite the FESS, SCS, and DUP treatment, the T&T olfactometry score and LMS remained abnormal; however, both improved four months after initiating TEZ. TEZ also lowered airway biomarkers, including the FeNO level. SCS: systemic corticosteroid; DUP: dupilumab; TEZ: tezepelumab; FESS: functional endoscopic sinus surgery; LMS: Lund–Mackay score; FeNO: fractional exhaled nitric oxide; NPS: nasal polyp score; SNOT-22: Sino-Nasal Outcome Test-22; PBE: peripheral blood eosinophil; IgE: immunoglobulin E; %FEV1: percent predicted forced expiratory volume in 1 s; T&T: T&T olfactometer; PSL: prednisolone

He was referred to the Department of Otolaryngology for nasal obstruction and anosmia and was diagnosed with CRSwNP. The nasal polyp score (NPS) was 7 (Figure 1), and the olfactory assessment score, using a T&T olfactometer (Daiichi Yakuhin Sangyo Co., Ltd., Tokyo, Japan), the standard test in Japan [5], was 5.8 (range, -1.0 to 5.8), indicating anosmia (i.e., a score ≥5.6) (Figure 1). Computed tomography (CT) showed pansinus opacification with a Lund-Mackay score (LMS) of 20 (range, 0-24) (Figure 1). After achieving temporary asthma control, bilateral FESS was performed, including wide maxillary antrostomy, total ethmoidectomy, sphenoidotomy, and frontal sinusotomy, with the preservation of the inferior, middle, and superior turbinates.

**Figure 2:**
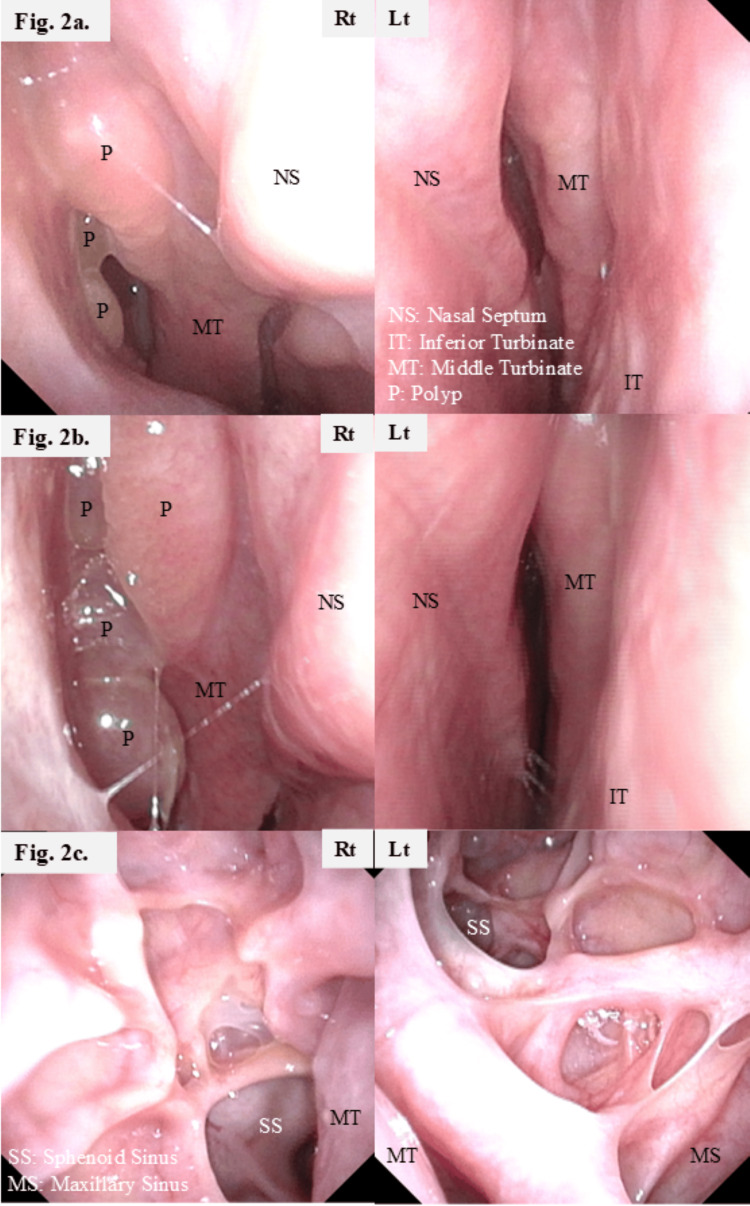
Endoscopic findings (a) before DUP administration, (b) five months after DUP administration, and (c) four months after TEZ administration a: a polyp is observed in the right middle meatus, along with mucosal edema; b: the polyp in the right middle meatus has increased in size; c: no polyp lesions are observed in the middle meatus, and mucosal edema has improved. DUP: dupilumab; TEZ: tezepelumab; NS: nasal septum; IT: inferior turbinate; MT: middle turbinate; P: polyp; SS: sphenoid sinus; MS: maxillary sinus

**Figure 3:**
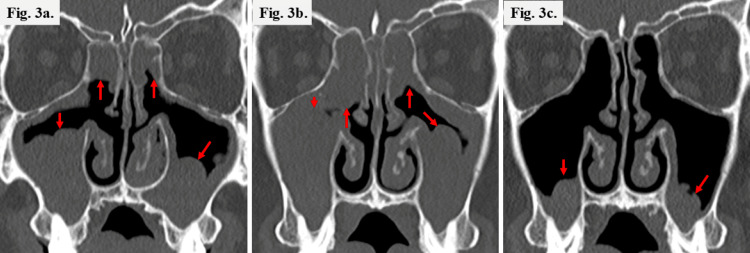
CT findings (a) before DUP administration, (b) five months after DUP administration, and (c) four months after TEZ administration a: soft tissue density is observed in the maxillary and ethmoid sinuses (red arrows); b: despite five months of DUP therapy, paranasal sinus opacification (red arrows) has further worsened; c: overall paranasal sinus opacification has resolved with only a faint residual shadow remaining on the floor of the maxillary sinus (red arrows). CT: computed tomography; DUP: dupilumab; TEZ: tezepelumab

However, nasal symptoms and asthma worsened within one month after surgery, necessitating daily SCS administration (10 mg prednisolone) for asthma control. Two months after surgery, a nasal polyp recurred in the right middle meatus (Figure 2a), and the LMS was 13 (Figures 1, 3a). His asthma remained poorly controlled with ongoing SCS dependence; therefore, three months after surgery, the Department of Respiratory Medicine initiated subcutaneous DUP (300 mg every two weeks). After initiating biologic therapy, the patient was followed according to a predefined safety and disease-monitoring schedule. Blood tests, nasal endoscopy, and pulmonary function tests were performed at one, two, four, and six months, and every six months thereafter. Olfactory testing and CT from the nasal cavity to the lung fields were performed at four and 12 months and annually thereafter. Clinical monitoring for fever, respiratory symptoms, and other adverse events was undertaken at each visit, with additional chest radiography performed when clinically indicated. Although asthma control improved slightly, SCS could not be discontinued. Five months after DUP initiation, the NPS was 4 (range, 0-8) (Figures 1, 2b), the T&T remained 5.8 (Figure 1), and the LMS was 17 (Figure 3b). Because asthma and nasal symptoms remained poorly controlled five months after DUP initiation, the Department of Respiratory Medicine switched the systemic therapy to TEZ (210 mg every four weeks). TEZ was initiated on the day the next DUP dose was scheduled, with no formal washout period between biologics. Four months after initiating TEZ, the NPS improved to 2 (Figures 1, 2c); the LMS decreased to 6 (Figures 1, 3c); the T&T olfactometry score improved to 2.6, indicating moderate hyposmia (range, 2.6-4.0) (Figure 1); and Sino-Nasal Outcome Test-22 decreased to 7 (range, 0-110) (Figure 1). One year after TEZ initiation, the T&T olfactometry score further improved to 2.4, indicating mild hyposmia (range, 1.1-2.5) (Figure 1). Importantly, asthma control was sufficiently improved with no need for SCS bursts.

## Discussion

TEZ is a human monoclonal antibody that blocks the activity of TSLP and acts upstream in the inflammatory cascade, thereby inhibiting the production of all type 2 cytokines. The WAYPOINT study [3], a recent phase 3 clinical trial, showed that TEZ significantly reduced the NPS, nasal congestion score, loss of smell score, Sino-Nasal Outcome Test-22 (SNOT-22) score, LMS, and total symptom score in patients with CRSwNP. However, to the best of our knowledge, this case report is the first to describe a favorable outcome following a switch from DUP to TEZ in a patient with CRSwNP refractory to DUP.

TEZ may benefit patients with CRSwNP who are refractory to, or ineligible for, DUP treatment. Our patient had marked improvement in upper and lower airway symptoms, with consistent reductions in nasal polyp burden, improved olfactory function, and decreased type 2 inflammation markers, as summarized in Figure 1. TSLP has been reported to be highly expressed in both eosinophilic and non-eosinophilic nasal polyps [6], with elevated expression at both the mRNA and protein levels in nasal polyps of patients with CRSwNP, regardless of eosinophil counts [7]. These findings suggest that TEZ is effective in various types of nasal polyps, including cases with an inadequate response to biologics targeting type 2 cytokines such as DUP.

TEZ may control steroid-refractory CRSwNP by targeting upstream mediators of type 2 inflammation, such as group 2 innate lymphoid cells (ILC2s), the major producers of IL-5 and IL-13, thereby contributing to steroid-resistant inflammation. ILC2s have been reported to contribute to the persistence of airway eosinophilia in severe asthma by producing type 2 cytokines in a localized and steroid-insensitive manner, even under high-dose SCS treatment [8]. Therefore, in steroid-refractory CRSwNP, as illustrated in this case, TEZ may represent an effective therapeutic option by modulating ILC2 activity and consequently attenuating type 2 inflammation.

TEZ may offer promising benefits for patients with CRSwNP complicated by severe asthma. A previous study demonstrated that significantly greater numbers of total and type 2 cytokine-producing ILC2s were detected in the blood and sputum of patients with severe asthma compared to those with mild asthma. Notably, in the sputum of patients with severe asthma, ILC2s were identified as the predominant cellular source of type 2 cytokines, such as IL-5 and IL-13 [8]. Given this evidence, therapies that reduce ILC2 activation, such as TEZ, may be beneficial for patients with CRSwNP complicated by severe asthma.

This report had some limitations. First, as a single case report, it cannot establish a causal relationship between the switch to TEZ and the observed clinical improvements, which may in part reflect natural fluctuations of disease activity. Second, because we did not measure ILC2s or TSLP levels, the mechanistic interpretation remains speculative. Third, the duration of DUP therapy (five months) may have been insufficient to fully assess its therapeutic potential. Fourth, temporal and treatment-related confounding cannot be excluded, as improvements may have been influenced by the passage of time and other concurrent interventions. Finally, the follow-up period was relatively short, limiting our ability to evaluate the long-term durability and safety of TEZ in this context.

## Conclusions

This case illustrates that TEZ may represent a therapeutic option for patients with CRSwNP and comorbid severe asthma who show an inadequate response to DUP. By targeting TSLP and modulating upstream inflammatory pathways, TEZ could provide broader clinical benefits, including in diseases that remain difficult to control despite corticosteroids or other biologics; however, these observations are hypothesis-generating and should not be interpreted as evidence of therapeutic superiority over existing agents. Larger, controlled studies with detailed biomarker assessment are required to confirm these findings and clarify the role of TEZ in the routine management of CRSwNP.
